# Computed tomography-based predictive nomogram for differentiating primary progressive pulmonary tuberculosis from community-acquired pneumonia in children

**DOI:** 10.1186/s12880-019-0355-z

**Published:** 2019-08-08

**Authors:** Bei Wang, Min Li, He Ma, Fangfang Han, Yan Wang, Shunying Zhao, Zhimin Liu, Tong Yu, Jie Tian, Di Dong, Yun Peng

**Affiliations:** 1 Department of Radiology, Beijing Children’s Hospital, Capital Medical University, National Center for Children’s Health, No.56 Nanlishi Road, Beijing, 100045 China; 2 Sino-Dutch Biomedical and Information Engineering School, Northeastern University, No. 3-11 Wenhua Road, Shenyang, China; 3 CAS Key Laboratory of Molecular Imaging, State Key Laboratory of Management and Control for Complex Systems, Institute of Automation, Chinese Academy of Sciences, No.95 Zhongguancun East Road, Beijing, 100190 China; 4 Department of Respiratory Medicine, Beijing Children’s Hospital, National Center for Children’s Health, Capital Medical University, No.56 Nanlishi Road, Beijing, 100045 China; 5 Beijing Advanced Innovation Center for Big Data-Based Precision Medicine, School of Medicine, Beihang University, No. 37 Xueyuan Road, Beijing, 100191 China; 6 University of Chinese Academy of Sciences, No.19 Yuquan Road, Beijing, China

**Keywords:** Child, Tuberculosis, Pulmonary, Pneumonia, Radiomics, Nomogram

## Abstract

**Background:**

To investigate the value of predictive nomogram in optimizing computed tomography (CT)-based differential diagnosis of primary progressive pulmonary tuberculosis (TB) from community-acquired pneumonia (CAP) in children.

**Methods:**

This retrospective study included 53 patients with clinically confirmed pulmonary TB and 62 patients with CAP. Patients were grouped at random according to a 3:1 ratio (primary cohort *n* = 86, validation cohort *n* = 29). A total of 970 radiomic features were extracted from CT images and key features were screened out to build radiomic signatures using the least absolute shrinkage and selection operator algorithm. A predictive nomogram was developed based on the signatures and clinical factors, and its performance was assessed by the receiver operating characteristic curve, calibration curve, and decision curve analysis.

**Results:**

Initially, 5 and 6 key features were selected to establish a radiomic signature from the pulmonary consolidation region (RS1) and a signature from lymph node region (RS2), respectively. A predictive nomogram was built combining RS1, RS2, and a clinical factor (duration of fever). Its classification performance (AUC = 0.971, 95% confidence interval [CI]: 0.912–1) was better than the senior radiologist’s clinical judgment (AUC = 0.791, 95% CI: 0.636-0.946), the clinical factor (AUC = 0.832, 95% CI: 0.677–0.987), and the combination of RS1 and RS2 (AUC = 0.957, 95% CI: 0.889–1). The calibration curves indicated a good consistency of the nomogram. Decision curve analysis demonstrated that the nomogram was useful in clinical settings.

**Conclusions:**

A CT-based predictive nomogram was proposed and could be conveniently used to differentiate pulmonary TB from CAP in children.

**Electronic supplementary material:**

The online version of this article (10.1186/s12880-019-0355-z) contains supplementary material, which is available to authorized users.

## Background

Pulmonary tuberculosis (TB) is one of the most widespread infections throughout the world and has a high incidence in developing countries. Pulmonary TB has a high morbidity and mortality [1], mostly in HIV-infected children [2]; however, it often occurs in children without HIV in endemic areas. It is essential for children with TB to be early diagnosed and appropriately treated with anti-TB drugs.

The positivity rate for pulmonary TB in children is less than 50% [2, 3] due to sampling challenges and its low bacterial load. The main challenge we experienced when diagnosing pulmonary TB in children relates to a lack of bacteriological confirmation. Currently, the diagnosis merely relies on an examination of clinical symptoms and radiological findings, which is not accurate enough. Sreeramareddy et al. [4] reported that the delayed time for TB diagnosis in China was 25–71 days. What is more, primary progressive pulmonary TB can present with more severe forms such as segmental or lobar consolidation (tuberculous pneumonia or caseous pneumonia) [5]. They are more common in children than in adults [6] and are accompanied with acute symptoms that are very difficult to distinguish from the respiratory infection in children. Zaro et al. [7] reported that hospitalized children with confirmed pulmonary TB presented acute/subacute symptoms, similar to the acute pneumonia in children. The nonspecific symptoms and signs of the pulmonary TB often overlap with the common pediatric pulmonary infections and especially with those of community-acquired pneumonia (CAP). The main pathogens that cause CAP in children include Streptococcus and Mycoplasma spp., of which Streptococcus spp. is the most common, accounting for 40% of CAP cases [8]. In Asian countries, 1–7% of cases presenting with CAP are re-diagnosed with pulmonary TB [9]. Therefore, it is essential to develop an effective tool for early differentiating pulmonary TB from CAP in children.

Radiomics is a novel tool adopting advanced image analysis algorithms that employ a large number of quantitative image features [10]. The integration of these features may generate powerful models to assist the disease diagnosis and prognosis [11, 12]. A number of articles have reported applications of radiomics in tumors, but few studies have reported on such applications in non-neoplastic disease.

In this study, we aimed to develop a computed tomography (CT)-based predictive nomogram for helping distinguish primary progressive pulmonary TB from CAP in children. We would also like to investigate the value of radiomics in non-neoplastic diseases.

## Methods

**Fig. 1:**
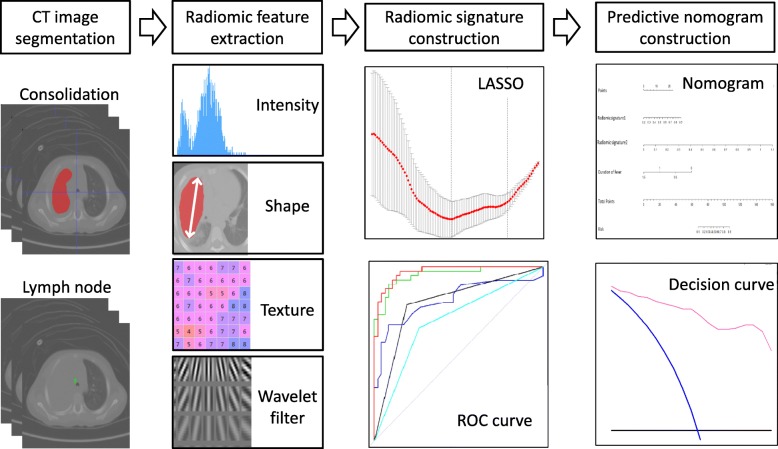
The workflow of this study. The pipeline of radiomics analysis includes CT image segmentation, radiomic feature extraction, radiomic signature construction, and predictive nomogram construction

This retrospective study was approved by the Ethics Committees of Beijing Children’s Hospital for using the data, and patient consent was waived. The workflow of our study is shown in Fig. 1.

### Patients

**Fig. 2:**
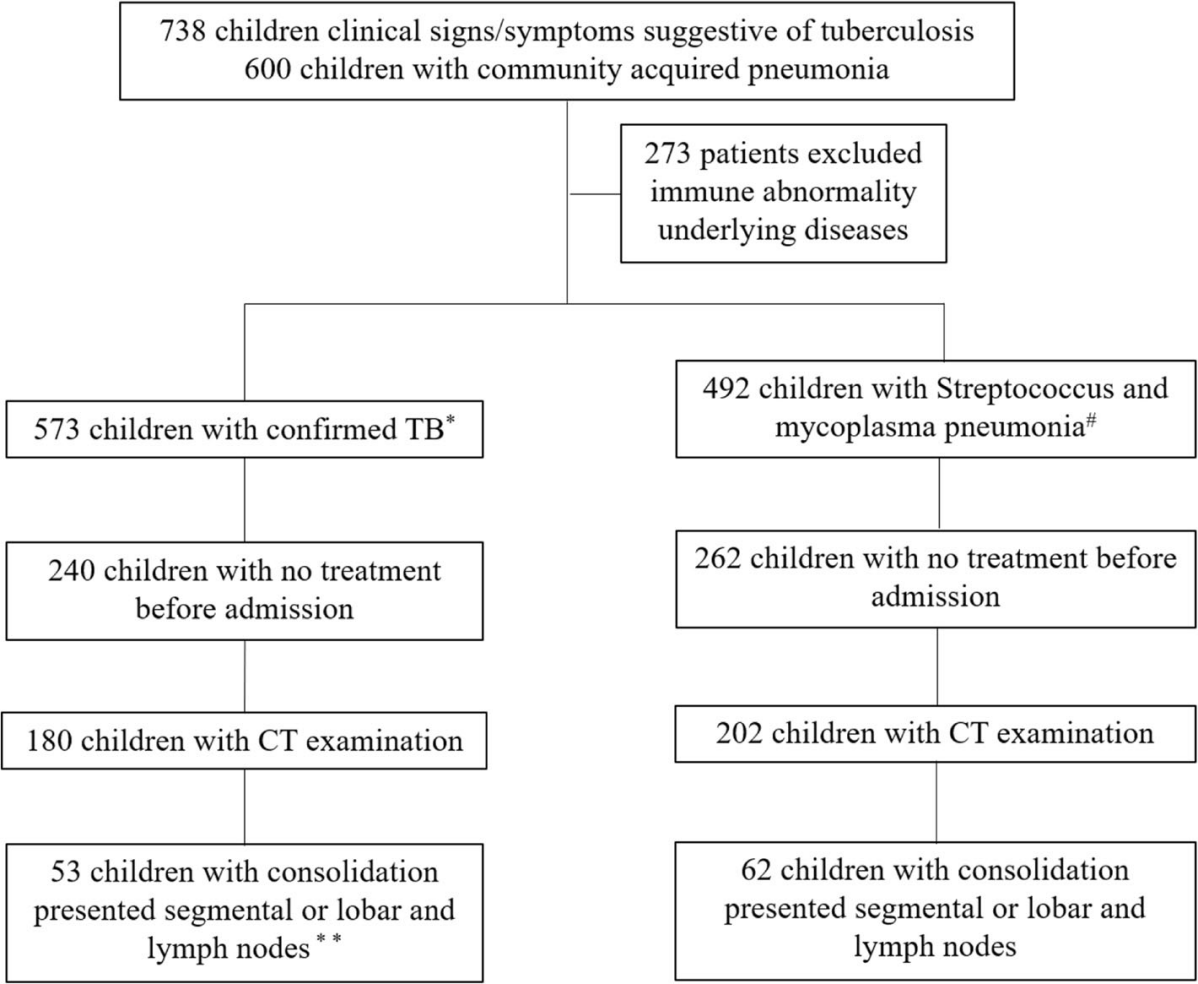
Patient recruitment in this study. Note: Confirmed TB* refers to *Mycobacterium tuberculosis* to be confirmed (culture or Xpert MTB/RIF assay) from at least one respiratory specimen (e.g., sputum, nasopharyngeal/gastric aspirate, and pleural fluid). Lymph nodes** refers to uniformity with no calcification and necrosis in the lymph nodes. Streptococcus and mycoplasma pneumonia^#^ are diagnosed via the detection of Streptococcus in pleural effusion or blood culture and positive IgM antibodies against Mycoplasma in the serum, respectively

**Fig. 3:**
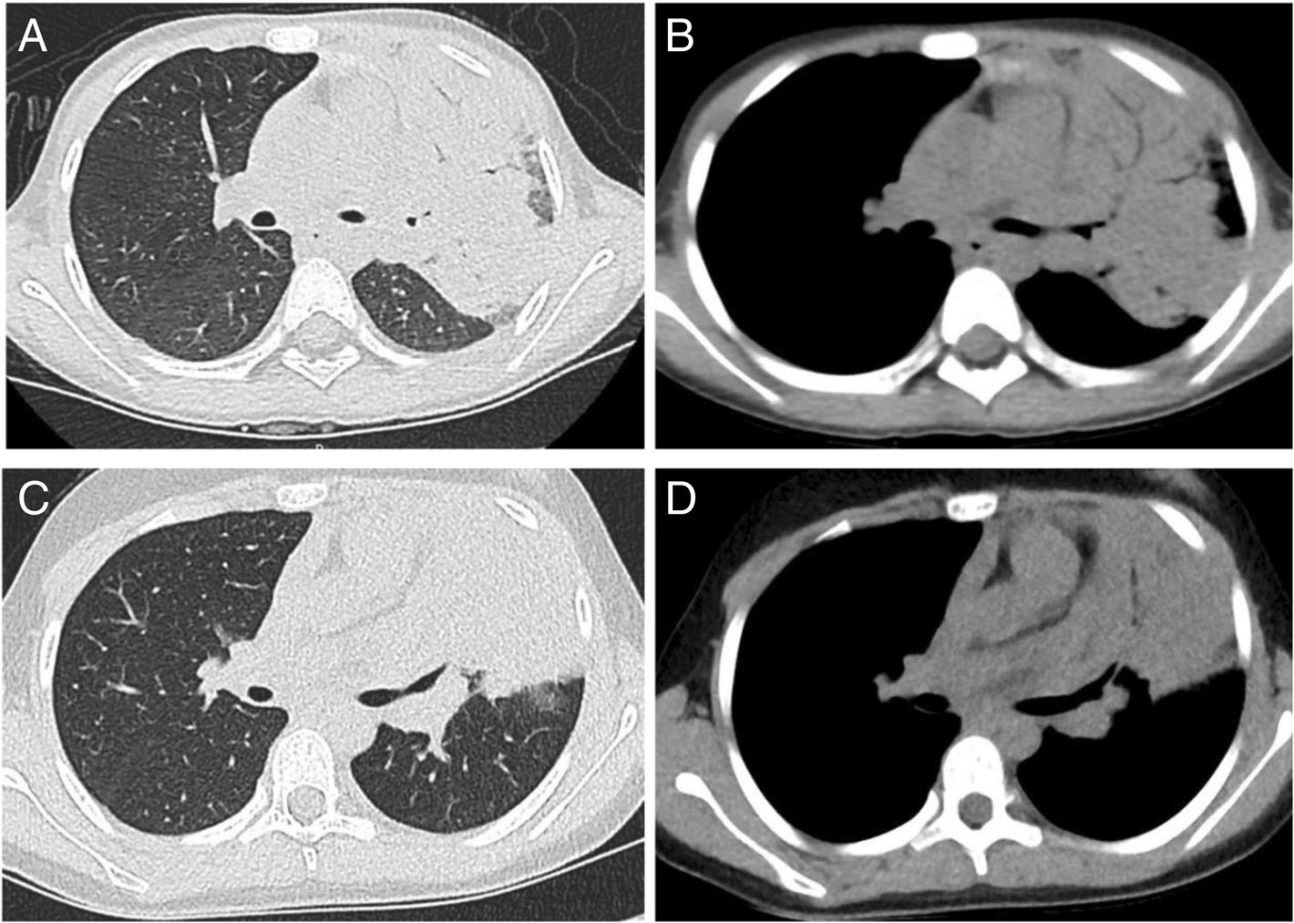
Examples of CT images from two patients with tuberculosis and pneumonia. (**a**) and (**b**) show the lung window and mediastinal window of the axial CT image of a 7-year-old girl with pulmonary TB in the left upper lobe. (**c**) and (**d**) show the lung window and mediastinal window of the axial CT image of a 10-year-old girl with CAP in the left upper lobe

Records for pulmonary TB and CAP patients attending our institution from January 2011 to January 2018 were obtained. The patient recruitment procedure is shown in Fig. 2. A total of 53 patients with pulmonary TB and 62 patients with CAP satisfied the inclusion criteria (Fig. 3) and were included in the study. We used two symptoms (pulmonary consolidation and mediastinal lymph nodes) to build a final predictive nomogram. Patients were grouped at random according to a 3:1 ratio: 86 patients in the primary cohort and 29 patients in the validation cohort. There were 51 male patients and 35 female patients in the primary cohort, the mean age was 4.01 ± 3.58 years, and an age range of 1-13 years. The validation cohort included 19 male patients and 10 female patients with a mean age of 2.28 ± 2.58 years and an age range of 0–10 years.

### CT examinations

All patients underwent an unenhanced low-dose CT examination of the chest on a 64-slice Discover CT750HD scanner (GE Healthcare, Waukesha, WI, USA). The area of coverage extended from the thoracic inlet to the diaphragm. Following institutional guidelines of the low-dose CT scan protocol, all low-dose thoracic CT studies were performed using specified parameters (5 mm section thickness, 100 kVp tube voltage, automatic tube current modulation technique, and a helical pitch of 1.375) to achieve an image noise index of 11-13HU. The radiation dose for the patients was 1.67 ± 0.83 mGy in CT dose index volum (CTDIvol) and 41.54 ± 22.78 mGy*cm in dose length product (DLP).

### CT image segmentation

For CT image segmentation, we used an axial mediastinum window archived under the Picture Archiving and Communication System (PACS, Carestream, Vaughan, ON, Canada) for digital imaging without preprocessing or normalization.

CT images were exported to ITK-SNAP software (Version 2.2.0; http://www.itksnap.org) for manual segmentation. A radiologist with 10 years of experience performed manual segmentation in an axial mediastinal window of unenhanced CT image using a three-dimensional region of interest (ROI) to delineate the margins of pulmonary consolidation (ROI1) and mediastinal lymph nodes (ROI2). For each patient, we examined lymph nodes positioned behind the superior vena cava for delineation of ROI2. Segmentation was verified by a senior radiologist with 15 years of experience.

### Data analysis

#### Radiomic feature extraction and Radiomic signature construction

Three-dimensional radiomic features were extracted from ROIs of the pulmonary consolidations and mediastinal lymph nodes, and each group included 485 features. These 485 features were divided into 4 categories: (a) shape and size features, (b) gray intensity features, (c) texture features, and (d) wavelet features [13]. Shape and size features reflect the phenotype of the ROIs, including the shape, area, volume, and level of compactness. Gray intensity features show differences in gray histograms and gray distributions of the ROIs. Texture features reveal the regularity of voxel relationships within the ROIs. Wavelet features reflect the transformation of gray intensity of texture features. The feature extraction method is described in detail in Additional file 1 (Appendix A1: CT feature extraction). Feature extraction was executed using MATLAB software (version 2014a; Mathworks, Natick, MA). The least absolute shrinkage and selection operator (LASSO) method is suitable for dimensionality reduction of high-dimensional data and is often used to extract the most useful features in previous studies [14].

We used the LASSO method to select key features from the radiomic features on primary cohort and built two radiomic signatures (RS1 and RS2) from ROI1 and ROI2, respectively. Then we validated the performances of the two signatures on the validation cohort. We also constructed a radiomic model by combining the two radiomic signatures.

#### Establishment of the predictive nomogram

Univariate analysis was used to select significant clinical factors with *p*-values < 0.05. Then, linear support vector machine (SVM) was used to build a predictive nomogram based on the radiomic signatures and significant clinical factors on the primary cohort. The SVM method is a generalized linear classifier for the binary classification of data in supervised learning that is widely used for pattern recognition purposes (e.g., face recognition and text categorization). In this study, The SVM is modeled using a linear kernel [15].

#### Performance evaluation of predictive nomogram

The performance of the predictive nomogram was evaluated on both the primary and validation cohorts. The receiver operating characteristic (ROC) curve was plotted to validate the classification ability, and calibration curves along with Hosmer-Lemeshow tests were performed to assess the goodness-of-fit of the nomogram.

For comparison, a senior radiologist (Y.W. with 6 years of experience) and a junior radiologist (T.Y. with 15 years of experience) independently reviewed the CT images with clinical information and reached final diagnosis. The clinical judgments by radiologists were also evaluated using the area under the ROC curve (AUC) value.

#### Clinical use

By quantifying the net benefit to the patient under different threshold probabilities, the clinical application value of the nomogram was determined through the decision curve analysis.

#### Statistical analysis

Statistical analysis was performed using R software (version3.3.4; http://www.Rproject.org). A two-sided *p*-value < 0.05 was used to indicate statistical significance. The glmnet package was used to implement the LASSO regression analysis. The pROC package was used to construct the ROC curve.

Univariate analysis was used to estimate the relationship between each patient’s clinical factors and the identification of the two diseases. Independent *t*-tests or Mann-Whitney U continuous variable tests were used to assess the differences in patient variables across the groups, and Fisher’s exact tests or chi-square tests were applied for categorical variables.

All methods were assessed using the ROC curves and compared by the AUCs along with the DeLong test. The point corresponding to the maximal Youden's index on the ROC curve of the primary cohort was used as the optimal threshold value and was also applied to the validation cohort. Sensitivity and specificity were calculated to assess the model performance.

Parts of the codes used in the study are shown in the Additional file 1: Appendix code.

## Results

### Clinical factors

Clinical factors (gender, age, hemoptysis, cough, fever, expectoration, white blood cell (WBC) count, and C-creative protein (CRP)) were found not significantly different between the two diseases, while the duration of fever was found significantly associated with the two diseases according to the univariate analysis (*p* < 0.05, Table 1) on the primary and validation cohorts. The probability of a patient suffering from pulmonary TB and CAP was not significantly different between the two groups (*p* = 0.962).

**Table 1:** Characteristics of patients in the primary and validation cohorts

Characteristic	Primary cohort			Validation cohort		
	CAP	Pulmonary TB	*p* value	CAP	Pulmonary TB	*p*-value
Gender, No. (%)			0.151			0.047*
Male	25 (54.35)	26 (65.00)		8 (50.00)	11 (84.62)	
Female	21 (45.65)	14 (35.00)		8 (50.00)	2 (15.38)	
Age, mean ± SD, years	4.51 ± 3.44	3.41 ± 3.66	0.181	2.69 ± 3.10	1.77 ± 1.62	0.332
Hemoptysis, No. (%)			0.083			–
+	0 (0.0)	3 (7.50)		0 (0.00)	0 (0.00)	
-	46 (100.0)	37 (92.50)		16 (100.00)	13 (100.00)	
Cough, No. (%)			0.324			0.82
-	2 (4.35)	4 (10.00)		0 (0.00)	3 (23.08)	
+	44 (95.65)	36 (90.00)		16 (100.00)	10 (76.92)	
fever, No. (%)			0.143			–
-	1 (2.17)	4 (10.00)		9 (56.25)	5 (38.46)	
+	45 (97.83)	36 (90.00)		7 (43.75)	8 (61.54)	
Expectoration, No. (%)			0.129			0.358
-	20 (43.48)	24 (60.00)		6 (37.50)	6 (46.15)	
+	26 (56.52)	16 (40.00)		10 (62.50)	7 (53.85)	
duration of fever (days)			<0.001***			<0.001
<10	43 (93.48)	15 (37.50)		15 (93.75)	3 (23.08)	
≥10	3 (6.52)	25 (62.50)		1 (6.25)	10 (76.92)	
WBC (*10^9/L), No. (%)			0.247			0.264
Median(IQR)	8.42 (6.70-10.16)	8.40 (7.10-11.41)		14.98 (14.18-17.26)	16.89 (16.30-19.77)	
Normal	27 (58.70)	20 (50.00)		5 (31.25)	5 (38.46)	
Abnormal	19 (41.30)	20 (50.00)		11 (68.75)	8 (61.54)	
CRP (mg/L), No. (%)			0.365			0.381
Median (IQR)	23.30 (11.53-56.25)	15.70 (9.44-33.88)		28.25 (16.00-71.75)	29.00 (14.00-53.60)	
Normal	7 (15.22)	8 (20.00)		1 (6.25)	3 (23.08)	
Abnormal	39 (84.78)	32 (80.00)		15 (93.75)	10 (76.92)	
Radiomic signature1 median	0.53 (0.32 to 0.86)		<0.001***	0.53 (0.33 to 0.74)		0.018*
Radiomic signature2 median	0.54 (0.12 to 0.98)		<0.001***	0.54 (0.20 to 1.26)		<0.001***

NOTE. The *p*-value was derived from univariable association analyses of each characteristic and of the two diseases. * denotes *p*-value< 0.05; ** denotes p-value< 0.01; *** denotes *p*-value<0.001

Abbreviations: *WBC* white blood cell, *CRP* C-reaction protein

### Construction of the Radiomic signature

A total of 970 radiomic features were extracted from the CT images (485 features from pulmonary consolidation regions and 485 from lymph node regions). The LASSO regression graph of these radiomic features is shown in Additional file 1 (Appendix Figure S1: The process of radiomic features selection using LASSO regression for RS1 and RS2) where key features for building the radiomic signatures are presented. Eleven key features highly related with the identification of the two diseases in the primary cohort were selected (*p* < 0.05, Table 2). Shape features such as “Surface_to_volume_ratio” calculates the surface area to volume ratio of the ROI, which describes the sphericity of the lesion, with lower values indicating a more compact spherical shape. First-order statistical feature “fos_maximum” and “fos_minimum” calculates the maximum and minimum grayscale intensities of the image, and describes the brightest and darkest image information of the image. Texture features such as “LRE” are calculated by the distribution of the image grayscale run matrix. The larger value of the LRE, the coarser of the texture in the ROI. Five features were extracted from the consolidation region (ROI1) and merged as a radiomic signature RS1. The other 6 features were extracted from the lymph node region (ROI2) and merged as a radiomic signature RS2. Significant differences of the radiomic signatures between pulmonary TB and CAP groups were found in both the primary cohort and the validation cohorts (*p* < 0.01, Table 1). A radiomic model was also built merging RS1 and RS2. The calculation formula of RS1 and RS2 are shown in Additional file 1 (Appendix A2: Radiomic signatures calculation formula).

**Table 2:** Radiomic feature selection results based on LASSO

Region	Features	Group	Filters	*p*-value
	X7_fos_maximum	Intensity	*X*_*HHL*_	<0.001***
	X0_GLCM_maximum_probability	GLCM	*X*_*LLL*_	0.008**
consolidation	X6_GLCM_IMC1	GLCM	*X*_*HLH*_	<0.001***
	X1_GLRLM_LRLGLE	GLRLM	*X*_*LLH*_	0.002**
	X1_GLRLM_LRE	GLRLM	*X*_*LLH*_	0.002**
lymph node	Max3D	Shape	NA	<0.001***
	Sph_dis	Shape	NA	<0.001***
	Compactness1	Shape	NA	<0.001***
	Surface_to_volume_ratio	Shape	NA	<0.001***
	X2_fos_minimum	Intensity	*X*_*HLH*_	<0.001***
	X0_GLRLM_LRHGLE	GLRLM	*X*_*HHL*_	<0.001***

NOTE. * denotes *p*-value< 0.05; ** denotes *p*-value< 0.01; *** denotes *p*-value< 0.001

### Predictive nomogram construction and validation

**Fig. 4:**
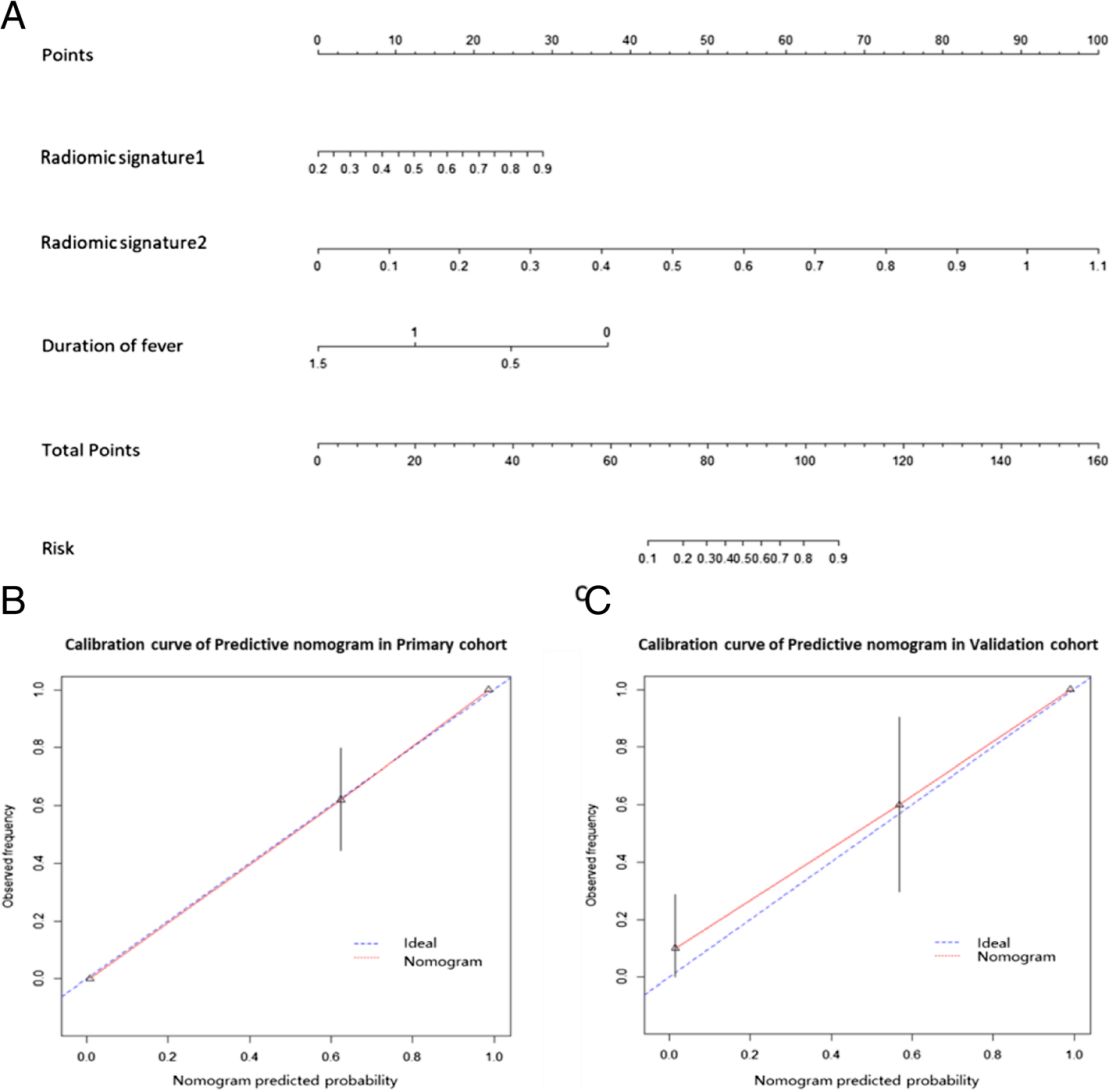
Construction and validation of predictive nomogram. (**a**) Predictive nomogram. (**b**) Calibration curve of the nomogram on primary cohort. (**c**) Calibration curve of the nomogram on validation cohort. The calibration curve demonstrates the agreement between the predicted risk by the nomogram and real outcomes. The 45-degree blue line represents a perfect prediction, and the red lines represent the predictive performance of the nomogram

**Fig. 5:**
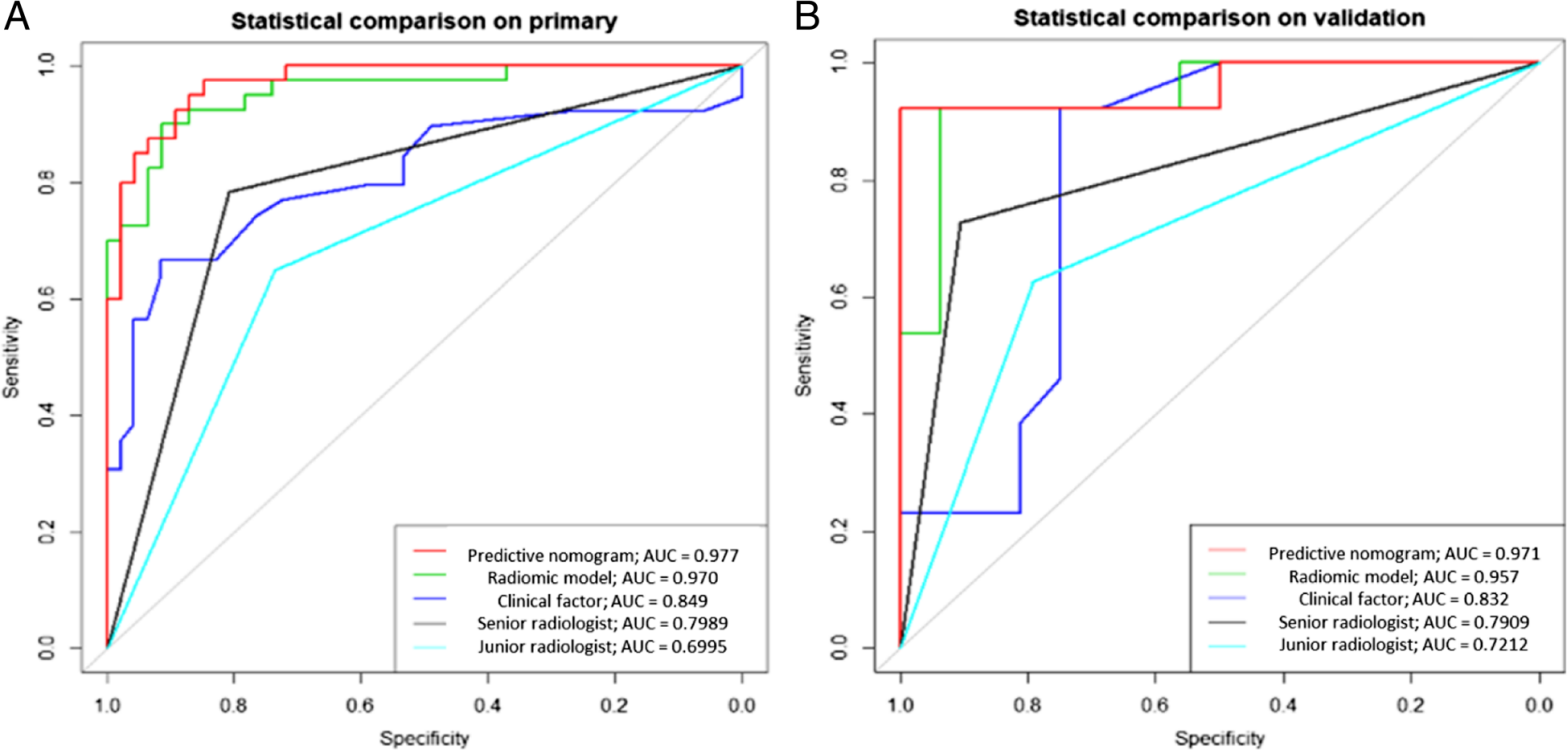
Receiver operating characteristic curve analysis of the models and radiologists’ diagnoses on the primary cohort (**a**) and validation cohort (**b**). The red, green, dark, black, and light blue lines denote the results of radiomic nomogram, radiomic model, clinical factors, a senior radiologist, and a junior radiologist, respectively

Two radiomic signatures (RS1 and RS2) and the duration of fever were identified as independent predictors of pulmonary TB and CAP. As shown in Fig. 4(a), a predictive nomogram was built by combining RS1, RS2, and duration of fever. The performances of RS1, RS2, radiomic model, clinical factor, and predictive nomogram are shown in Table 3. The predictive nomogram had the best differentiation ability of the two diseases with an AUC of 0.977 (95% CI, 0.953–1) on the primary cohort and an AUC of 0.971 (95% CI, 0.912–1) on the validation cohort, as shown in Fig. 5(a, b). In the primary cohort, the AUC value diagnosed by the senior radiologist was 0.799 (95% CI, 0.716-0.884), with an accuracy of 0.802 (95% CI, 0.711–0.872); and the AUC value diagnosed by the junior radiologist was 0.700 (95% CI, 0.602-0.797), with an accuracy of 0.698 (95% CI, 0.608–0.790). In the validation cohort, the AUC value diagnosed by the senior radiologist was 0.791 (95% CI, 0.636-0.946), with an accuracy of 0.793 (95% CI, 0.603-0.920); and the AUC value diagnosed by the junior radiologist was 0.721 (95% CI, 0.551-0.892), with an accuracy of 0.724 (95% CI, 0.528-0.873).

**Table 3:** Performances of RS1, RS2, radiomic model, clinical factor, and predictive nomogram

Index	Primary Cohort							Validation Cohort						
	RS1	RS2	radiomic model	clinical factors	predictive nomogram	senior radiologist	junior radiologist	RS1	RS2	radiomic model	clinical factors	predictive nomogram	senior radiologist	junior radiologist
TP	27	35	38	25	39	30	29	10	11	12	10	12	10	9
TN	41	42	42	43	41	39	31	14	15	15	15	15	13	12
FN	13	5	2	15	1	10	11	3	2	1	3	1	3	4
FP	5	4	4	3	5	7	15	2	1	1	7	1	3	4
Acc	79.07%	89.53%	93.02%	79.07%	93.02%	80.23%	69.77%	82.76%	89.66%	93.10%	86.21%	93.10%	79.31%	72.41%
Sen	0.675	0.875	0.950	0.625	0.975	0.750	0.725	0.769	0.846	0.923	0.769	0.923	0.769	0.692
Spe	0.891	0.913	0.913	0.935	0.891	0.848	0.673	0.875	0.938	0.938	0.938	0.938	0.813	0.750
PPV	0.844	0.897	0.904	0.893	0.886	0.811	0.695	0.833	0.917	0.923	0.909	0.923	0.769	0.692
NPV	0.759	0.894	0.955	0.741	0.976	0.796	0.738	0.824	0.882	0.938	0.833	0.938	0.813	0.750
AUC (95%CI)	0.837 (0.753–0.921)	0.948 (0.903–0.994)	0.970 (0.939–1)	0.849 (0.769–0.929)	0.977 (0.953–1)	0.799 (0.716-0.884)	0.700 (0.602-0.797)	0.803 (0.615–0.990)	0.938 (0.846–1)	0.957 (0.889–1)	0.832 (0.677–0.987)	0.971 (0.912–1)	0.7910.636-0.946)	0.721 (0.551-0.892)

NOTE. *TP* true positive, *TN* true negative, *FN* false negative, *FP* false positive, *Acc* accuracy, *Sen* sensitivity, *Spe* specificity, *PPV* positive predictive value, *NPV* negative predictive value, *AUC* area under ROC curve, *CI* confidence interval

The calibration curves of the nomogram in Fig. 4(b, c) showed that the predictions agreed well with the observations. The Hosmer-Lemeshow test results were not significant (*p* > 0.05), indicating no deviation from a perfect fit.

### Clinical use

**Fig. 6:**
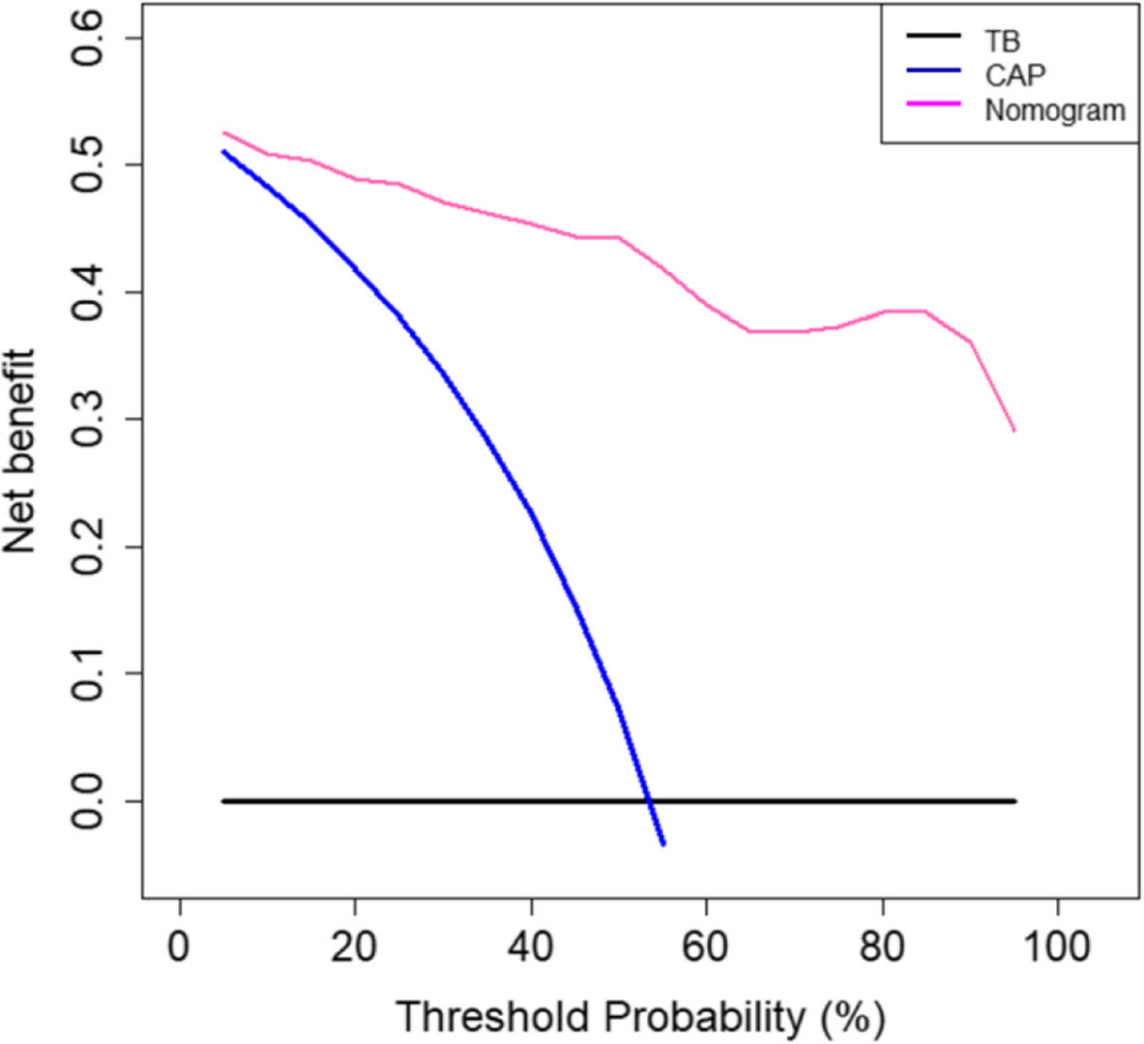
Decision curve analysis of the predictive nomogram. The x-axis and y-axis represent the threshold probability value and the net benefit, respectively. The red, blue, and black lines represent the treatment benefits using the nomogram, treating all patients as CAP, and treating all patients as pulmonary TB

Figure 6 illustrates the decision curve analysis of the predictive nomogram. The threshold probability level is the point at which the expected benefit of treatment is equal to the expected benefit of avoiding treatment. Our nomogram showed better treatment benefit than both “treating all patients as CAP” and “treating all patients as pulmonary TB” strategies.

## Discussion

To our knowledge, no previous study has analyzed cases of primary progressive pulmonary TB and CAP using radiomics. In our study, the predictive nomogram was found to be more effective than radiomic signatures of pulmonary consolidation/lymph nodes or clinical factors alone. Moreover, the diagnostic accuracy of the predictive nomogram was better than the radiologists’ subjective judgments. The predictive nomogram was based on routine CT scan and clinical factor, which was easy to use in the clinical practice. Therefore, this predictive nomogram may serve as a potential tool for distinguishing these two major pulmonary diseases in children.

Nambu [16] demonstrated that pulmonary TB could manifest as CAP. In the early diagnostic stage, it is difficult to distinguish the pulmonary TB from CAP. In our study, only roughly 75.47% of pulmonary TB cases were correctly diagnosed in the entire cohort by senior radiologist, echoing the results of previous studies conducted in Iran [17], Hong Kong [18], and Singapore [19]. Typical CT manifestations of pulmonary TB [20] include centrilobular nodules, cavities, lymph nodes containing calcification densities, and caseous necrosis. However, in the present study, all children with pulmonary TB only exhibited segmental or lobar pulmonary consolidation and lymph nodes on unenhanced CT images without any typical CT features of pulmonary TB. These CT manifestations are similar to those of common CAP with lung lobar distribution. It is thus highly challenging to differentiate the two diseases via visual assessment. Moreover, the CRP values of patients with pulmonary TB were found significantly higher than normal in this study; these CRP values were similarly elevated in patients with CAP [21]. The radiologists were also unable to get more useful information through laboratory examinations. Thus, the diagnostic rate achieved by the radiologists was lower than that achieved by the predictive nomogram.

The field of radiomics has demonstrated its potential capacity to capture useful information using machine learning methods and to enhance the accuracy of clinical differential diagnosis. In our study, 970 candidate features were extracted from CT images and were reduced to only 11 potential predictors by using a LASSO regression model to develop the radiomic signatures. The 11 radiomic features derived from pulmonary consolidation and lymph nodes were divided into four types (shape, texture, gray intensity features, and wavelet features) and varied significantly between cases of primary progressive pulmonary TB and CAP. Lymph nodes are complex in structure and contain microscopic textural features from unenhanced CT images but imperceptible to the naked eye. In this study, Max3D, Sph_dis, Compactness1, and Surface_to_volume_ratio parameters were obtained from shape features, which described the overall shapes and sizes of lymph nodes or other properties of lymph node outlines. These features were all associated with the diagnosis of the two diseases.

In our study, texture (GLCM and GLRLM) and gray intensity features extracted from the pulmonary consolidation and lymph nodes were significant radiomic features of the two diseases, but an exact clinical explanation for this remains undetermined. According to a previous study, the features often capture textural variations to quantify the spatial relationships of voxels within an image. For example, they can quantify voxels when they present similar values (e.g., related to necrosis) or spatial variations (e.g., related to intratumor heterogeneity) [22]. In our study, the Long Run Emphasis (LRE) of the texture feature was significantly greater in cases of pulmonary TB, presumably reflecting the coarser structural textures of pulmonary consolidation when compared with lesions of CAP. In clinical cancer research, the texture features were proven to reflect the image heterogeneity of the tumor [23, 24], and thus indicated the genetic heterogeneity and invasiveness of the tumor. We speculate that the image heterogeneity of pulmonary consolidation and lymph nodes varies between pulmonary TB and CAP.

However, this study still presents some limitations. As a retrospective study, most cases of CAP were not subjected to enhanced CT examination. Therefore, the manual delineation of lymph node ROIs was subject to the experience of the radiologists, which may have affected the accuracy of the results. Given the patients’ strict inclusion criteria, the sample size was small, which may have affected the model’s reliability, and future studies should utilize larger sample size. In addition, we only examined cases involving pulmonary consolidation and lymph nodes. Other common pulmonary TB, for example with “tree bud” sign [25], should be further studied.

## Conclusion

In conclusion, we proposed a CT-based predictive nomogram to differentiate primary progressive pulmonary TB and CAP. The CT-based predictive nomogram could serve as a new differential diagnostic tool for pulmonary infection diseases for pediatricians and radiologists.

## Additional file

Additional file 1: (DOCX 259 kb)

## Data Availability

The datasets used and/or analyzed during the current study available from the corresponding author on reasonable request.

## Abbreviations

CAP: Community-acquired pneumonia
CT: Computed tomography
LASSO: Least absolute shrinkage and selection operator
LRE: Long Run Emphaisi
ROC: Receiver operating characteristic
ROI: Region of interest
RS1: Radiomic signature from pulmonary consolidation
RS2: Radiomic signature from lymph node
SVM: Support Vector Machine
TB: Pulmonary tuberculosis
