# Supplementary material for: Computed tomography-based predictive nomogram for differentiating primary progressive pulmonary tuberculosis from community-acquired pneumonia in children
Source: BMC Med Imaging. 2019 Aug 8;19:63. doi: 10.1186/s12880-019-0355-z (PMC6688341; doi:10.1186/s12880-019-0355-z)
Supplement: Supplementary file 1 — (DOCX 259 kb) [file 12880_2019_355_MOESM1_ESM.docx]

**Appendix**

**Appendix A1: CT feature extraction**

In this study, a total of 485 CT radiomic features were extracted from pulmonary consolidation region per patient. The same 485 features were also extracted from the lymph node region. Feature extraction was implemented using MatLab 2014a (MathWorks, Natick, MA, USA). The 485 features were divided into four types: first-order statistics features, shape- and size-based features, textural features, and features after wavelet transform. The formula of these features were the same with our previous study [1].

Using a least absolute shrinkage and selection operator (LASSO) algorithm, we selected the 11 features that were most associated with pulmonary tuberculosis. The features were as follows:

***X7_fos_maximum****:* The maximum gray level intensity within the ROI:

maximum=max(X)

***X0_GLCM_maximum_probability:*** Maximum Probability is occurrences of the most predominant pair of neighboring intensity values:

maximum probability = max(p(i,j))

***X6_GLCM_IMC1:***

IMC1 = $\frac{HXY-HXY1}{max\{HX,HY\}}$

***X1_GLRLM_LRLGLE:*** LRLGLRE measures the joint distribution of long run lengths with lower gray-level values:

LRLGLRE = $\frac{\sum_{i=1}^{N_{g}} \sum_{j=1}^{N_{r}} \frac{P(i,j|\theta)j^{2}}{i^{2}}}{N_{Z}(\theta)}$

***X1_GLRLM_LRE:*** LRE is a measure of the distribution of long run lengths, with a greater value indicative of longer run lengths and more coarse structural textures:

LRE = $\frac{\sum_{I=1}^{Ng} \sum_{j=1}^{N_{r}} P(i,j|\theta)j^{2}}{N_{Z}}$

***Max3D:*** The shape and size feature that describes the maximum three-dimensional tumor diameter in the original image.

***Sph_dis:***

spherical disproportion =$\frac{A}{4\pi R^{2}}$ =$\frac{A}{\sqrt[3]{36\pi V^{2}}}$

***Compactness 1:*** Compactness 1 is a measure of how compact the shape of the tumor is relative to a sphere (most compact).

compactness 1 = $\frac{V}{\sqrt{\pi A^{3}}}$

***Surface_to_volume_ratio:*** Here, a lower value indicates a more compact (sphere-like) shape. This feature is not dimensionless, and is therefore (partly) dependent on the volume of the ROI:

surface to volume ratio = $\frac{A}{V}$

***X2_fos_minmum:***

minimum = min(X)

**Reference**:

[1] Zhu X, Dong D, Chen Z, Fang M, et al, Radiomic signature as a diagnostic factor for histologic subtype classification of non-small cell lung cancer, European Radiology, 2018, 28(7): 2772-2778.

**Appendix A2: Radiomic signatures calculation formula:**

Radiomic signature1 = X7_fos_maximum * 1.297e-01 + X0_GLCM_maximum_probability5.181e-02 + X6_GLCM_IMC1 *1.975e-02 - X1_GLRLM_LRLGLE * 1.282e-02 - X1_GLRLM_LRE * 4.178e-16

Radiomic signature2 = Compactness1 * 0.019 - Max3D * 0.019 - Sph_dis * 0.066 + Surface_to_volume_ratio * 0.121 + X2_fos_minimum * 0.015 - X0_GLRLM_LRHGLE * 0.002

Radiomic model = 5.942 * Radiomic signature1 + 12.388 * Radiomic signature2 – 9.417

**Appendix Figures:**

**
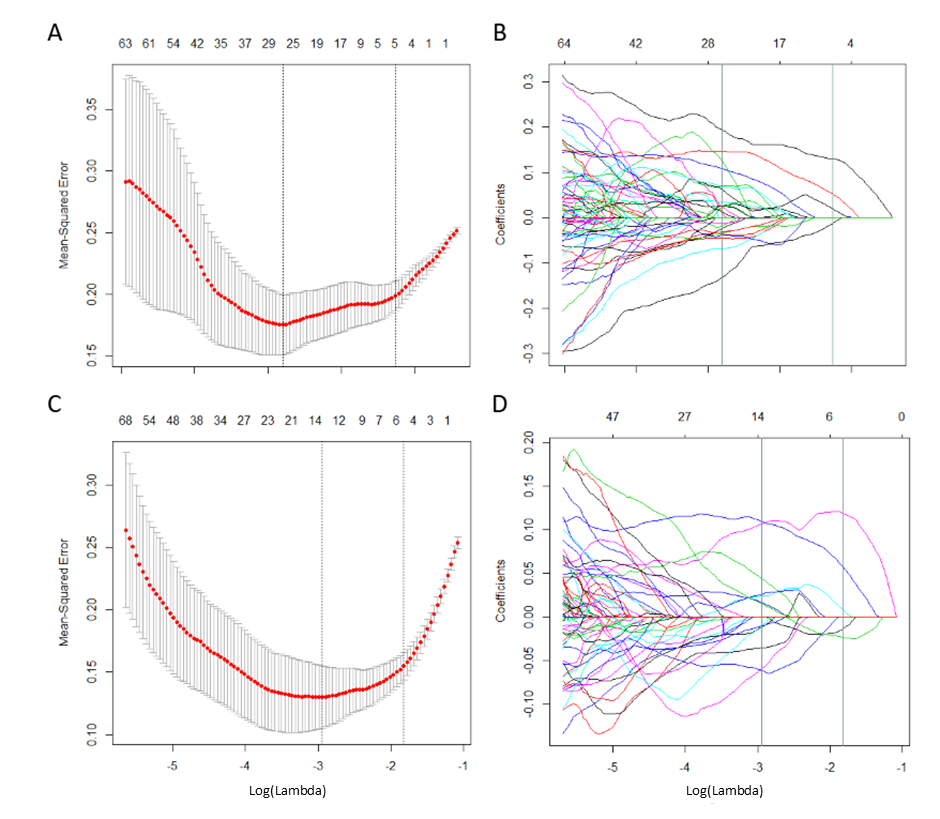
**

**Figure S1**. The process of radiomic features selection using LASSO regression for RS1 and RS2. (A) and (C) show the selection of the tuning parameter λ for RS1 and RS2, respectively. The selection of the tuning parameter for the LASSO regression was via 10-fold cross-validation based on minimum criteria. The upper and lower x-axis indicate the number of predictors and log(λ), while the y-axis indicates mean-squared error. The optimal log(λ) = -0.85 was automatically selected for RS1, while log(λ) = -0.77 was selected for RS2. (B) and (D) show the LASSO coefficient profiles of the selected features for RS1 and RS2, respectively. Finally, 5 and 6 radiomic features with nonzero coefficients are automatically selected for RS1 and RS2, respectively.

**Appendix code:**

Feature selection using LASSO:

library(glmnet)

g <- cv.glmnet(Train, StatusTrain, nfolds = 10)

plot(g)

g$lambda.1se

g.best <- g$glmnet.fit

g.coef <- coef(g$glmnet.fit, s = g$lambda.1se)

g.coef[which(g.coef != 0)]

SVM model:

svmfit <- svm(A ~X1+X1.1 , data = sPP, kernel = "linear", cost = 10, scale = FALSE) # linear svm, scaling turned OFF

summary(svmfit)

Multivariate regression analysis：

model <- glm(PP$StatusTrain ~.,family=binomial(link='logit'),data=PP)

summary(model)

Construction of monogram:

f1 <- lrm(A~ X1+X1.1, data = sPP,x=TRUE,y=TRUE)

nom <- nomogram(f1, fun= function(x)1/(1+exp(-x)),lp=F, funlabel="Risk")

plot(nom)
